# Assessment of the Educational Quality, Accuracy, and Transparency of WebSurg Videos on Minimally Invasive Video-Assisted Parathyroidectomy

**DOI:** 10.7759/cureus.18942

**Published:** 2021-10-21

**Authors:** Banu Yigit, Bulent Citgez

**Affiliations:** 1 General Surgery, Elazig Fethi Sekin City Hospital, Elazig, TUR; 2 General Surgery, Sisli Hamidiye Etfal Medical Practice and Research Center, University of Health Sciences, Istanbul, TUR

**Keywords:** websurg, video, score, quality, parathyroid, mivap

## Abstract

Background

The internet has changed the way both physicians and patients search for health information. WebSurg® is a valuable source of information that informs surgeons about new technologies and techniques and aims to promote quality, safety, and patient-centered care. In this study, our aim is to evaluate the popularity, quality, transparency, and accuracy of videos about minimally invasive video-assisted parathyroidectomy (MIVAP).

Materials and methods

A total of 31 videos related to MIVAP returned by the WebSurg® search engine in response to the keywords “MIVAP”, “video assisted parathyroidectomy’’, and “minimally invasive parathyroidectomy’’ were included in this study. Videos were evaluated in terms of time since upload, run time, country, academic degree, and the number of views and likes. The popularity of videos was determined by the video power index (VPI) formula. The DISCERN questionnaire score (DISCERNqs), global quality score (GQSc), and Journal of American Medical Association benchmark criteria (JAMABC) scoring systems were used to analyze WebSurg® videos for reliability and quality.

Results

The academic degree of the members was MD in 90.32% of uploaded videos. Forty-eight point thirty-eight percent (48.38%) of the videos were uploaded by members from France. There was no significant difference between the DISCERNqs, JAMABC, GQSc, and MIVAP scoring system (MIVAP-SS) scores in terms of academic degree and country. A statistically significant negative correlation was found between the time since upload and the VPI score (r=-0.683, p<0.001). The run time was positively correlated with the DISCERNqs, JAMABC, GQSc, and MIVAP-SS scores (p=0.003, p=0.002, p=0.003, p<0.001, respectively). For the MIVAP-SS score, the Spearman correlation analysis demonstrated a statistically significant positive correlation with VPI, DISCERNqs, JAMABC, and GQSc (p<0.05).

Conclusion

Videos about MIVAP are helpful for surgeons to learn the procedure step-by-step before the surgery they will be performing but still below the expected quality. It is recommended to use MIVAP-SS points, which is a novel scoring system, to ensure standardization and improve quality.

## Introduction

Improved preoperative imaging techniques and an intraoperative assay to confirm normalization of the parathyroid hormone have allowed changes in the treatment of hyperparathyroidism. The surgical difficulty of parathyroidectomy is due to the variability in the number and localization of parathyroid glands. Therefore, parathyroidectomy should be performed by surgeons with adequate training and experience. Surgeons should also choose a surgical approach that has a high cure rate, low-risk profile, and cost comparable to other available techniques. Since the first report of endoscopic parathyroidectomy by Gagner [1] in 1996, video-assisted techniques have been applied to surgery of the neck, and several series have documented the feasibility of these approaches for parathyroid diseases [2]. Minimally invasive video-assisted parathyroidectomy (MIVAP) was first described by Miccoli et al. in 1997 [3]. MIVAP is one of the most common targeted approaches for hyperparathyroidism, focused on the pathological parathyroid gland(s), and all patients with sporadic primary hyperparathyroidism are potential candidates for this technique. It provides the opportunity to explore both sides in case of controversial preoperative imaging and to operate on the associated thyroid lesion located on the opposite side of the parathyroid lesion [4].

Internet use is increasingly common, and it has become an indispensable part of our daily life. Besides, with the widespread use of the Internet, Research Institute against Digestive Cancer (IRCAD), the world-renowned higher education research center, was established in 1994 under the leadership of Professor Jacques Marescaux. The foundations of WebSurg®, IRCAD's free online university, were laid in 2000 [5]. It has become a reference website on minimally invasive surgical techniques and a communication network between surgeons and experts from all over the world. WebSurg® is beneficial in increasing the surgical knowledge and skills of surgeons who are working in a fast-paced environment and are in need of ubiquitous access to educational content [6]. Monitoring the images recorded with video-recording systems after the surgery increases the standardization and cohesion of the surgical team. Surgical procedures become safer and more effective with standardization. The main purpose of this study is to evaluate the popularity, quality, transparency, and accuracy of MIVAP videos on WebSurg® using the scoring systems of the DISCERN questionnaire score (DISCERNqs), global quality score (GQSc), Journal of American Medical Association benchmark criteria (JAMABC), video power index (VPI), and MIVAP scoring system (MIVAP-SS).

## Materials and methods

Online research of WebSurg® content was conducted by using the keywords “MIVAP”, ‘’video assisted parathyroidectomy’’, and ‘’minimally invasive parathyroidectomy’’ on July 1, 2021. A total of 31 videos about the MIVAP procedure were analyzed. Any online content related to both the surgical intervention and lecture was included in the research protocol. Descriptive characteristics of the videos, including time since upload, run time, number of views, number of likes, country, and academic degree, were examined. The transparency, educational quality, and reliability of the WebSurg® data were assessed through DISCERNqs, JAMABC, and GQSc. We additionally recorded the popularity of the WebSurg® content by using VPI [7-10]. The medical and technical quality of the data was evaluated through MIVAP-SS.

MIVAP-SS

MIVAP-SS, consisting of 25 criteria, was utilized for a more comprehensive appraisal of videos in terms of the MIVAP technique. We altered this scoring system from the recent literature about MIVAP surgery [11-15]. Scoring for each criterion was done independently by two general surgeons performing minimally invasive parathyroidectomy techniques in routine daily practice. The MIVAP-SS was used to conduct an independent assessment of MIVAP surgery by evaluating the medical and technical quality of the information. This scoring system has been modified based on factors affecting preoperative, perioperative, and postoperative outcomes specific to MIVAP surgery. The K-means clustering method for quality assessment of videos was applied according to MIVAP-SS scores, and they were divided into three groups as ideal, intermediate, and poor levels of quality (MIVAP-SS point >11.84, 7.17-11.84, <7.17, respectively) (Table 1).

**Table 1:** MIVAP scoring system MIVAP: minimally invasive video-assisted parathyroidectomy

Preoperative evaluation
1. Has the complete information been provided on how to select the appropriate patient for the operation?
2. Is it mentioned whether the patient has any comorbid disease?
3. Is the age of the patient specified on video?
4. Is the gender of the patient specified on video?
5. Is the information about the patient’s past surgical history stated?
6. Are patients’ symptoms specified on the video or if it is an asymptomatic patient, is it specified on the video?
7. Are the indications of surgical approach specified on video?
8. Is the side of the parathyroid (right, left, bilateral) specified on the video?
9. Are preoperative radiological imaging findings (ultrasound, sestamibi scintigraphy, etc.) specified on video?
Peroperative evaluation
1. Has the information been given about the patient’s position in the operation room?
2. Has the information been given about the kind of anesthesia or cervical block is used?
3. Has the information been given about the endotracheal intubation method?
4. Has the information been given about the position of the surgical team?
5. Has the information been given about the energy devices (Ligasure, Harmonic scalpel, etc.) to be used?
6. Is it specified what diameter endoscope will be used for the surgery?
7. Has the information been given about the endoscopic instruments (atraumatic spatulas, spatula-shaped aspirator, ear-nose-throat forceps, scissors, etc.)?
8. Has the information been given about the length of the skin incision?
9. Has the information been given about the dissection technique?
10. Has any information been given about the prominent landmarks (recurrent laryngeal nerve, inferior thyroid artery, etc.) that must be seen during the surgery?
11. Has the information been provided on which intraoperative localization technique (frozen section examination, intraoperative PTH, gamma probe, etc.) was used?
12. Has the information been provided on operative time?
Postoperative evaluation
1. Has the information been given about postoperative complications?
2. Has the information been given about the postoperative pain levels, and postoperative recovery?
3. Is the hospitalization period or discharge time specified on video?
4. Has the information been given about the postoperative cosmetic results and patients’ satisfaction?

Video power index

The popularity of the videos was assessed using VPI, which was first introduced by Erdem and Karaca [10]. The formula used to calculate the VPI: like ratio x view ratio/100, like ratio: number of likes x 100/number of views, view ratio: number of views x 100/time since upload (days).

DISCERN questionnaire

The sources of health information and the amount of available literature are increasing with the demand for more and better information about health problems and treatment options. The DISCERN questionnaire developed by Charnock et al. [16] was used to evaluate the reliability and information quality of the educational materials. The questionnaire consists of 16 questions, with a five-point scale for each question, yielding a minimum score of 16 and a maximum score of 80. Thirty to 64 points mean “partially fulfilled” (Table 2).

**Table 2:** DISCERN questionnaire

DISCERNqs
Are the aims clear?
Does it achieve its aims?
Is it relevant?
Is it clear what sources of information were used to compile the publication (other than the author or producer)?
Is it clear when the information used or reported in the publication was produced?
Is it balanced and unbiased?
Does it provide details of additional sources of support and information?
Does it refer to areas of uncertainty?
Does it describe how each treatment works?
Does it describe the benefits of each treatment?
Does it describe the risks of each treatment?
Does it describe what would happen if no treatment is used?
Does it describe how the treatment choices affect the overall quality of life?
Is it clear that there may be more than one possible treatment choice?
Does it provide support for shared decision-making?
Based on the answers to all of the above questions, rate the overall quality of the publication as a source of information about treatment choices.

GQSc

The GQSc, defined by Bernard et al. [8], is a five-point scale based on quality, flow-through, and ease of access to available information (Table 3).

**Table 3:** Global quality score (GQSc)

GQSc	
1-	Poor quality, not at all useful for patients
2-	Generally poor quality, very limited use to patients present
3-	Moderate quality, somewhat useful for patients
4-	Good quality, useful for patients
5-	Excellent quality, very useful for patients

JAMA benchmark criteria

Video reliability and transparency were assessed according to the JAMA benchmark criteria (JAMABC; score range 0-4), described by Silberg et al. [9]. Zero (0) points mean the criterion is not fulfilled, 1 point means insufficient fulfillment of the criterion, 2-3 points mean partially fulfilled, and 4 points mean completely fulfilled (Table 4).

**Table 4:** Journal of American Medical Association benchmark criteria (JAMABC)

JAMABC
authorship: authors and contributors, their affiliations, and relevant credentials should be provided
attribution: references and sources for all content should be listed clearly, and all relevant copyright information noted
disclosure: website ownership should be prominently and fully disclosed, as should any sponsorship, advertising, underwriting, commercial funding arrangements or support, or potential conflicts of interest
currency: dates that content was posted and updated should be indicated

Statistical analysis

Statistical analyses were performed with the Statistical Package version 17.0 (SPSS Inc., Chicago, IL). The normal distribution of quantitative data was tested with Kolmogorov-Smirnov and graphical evaluations. Descriptive statistical methods (mean, standard deviation, median) were used to evaluate the study data. The Kruskal Wallis test was used for non-normally distributed data to calculate the difference between the groups. The correlations between continuous variables were measured with the Spearman correlation test in accordance with the data distribution. The differences were considered to be statistically significant with p≤0.05.

## Results

The descriptive properties of the videos analyzed according to the academic title, country, time since upload, run time, number of views, and likes are given in Table 5.

**Table 5:** Descriptive statistics on the properties' characteristic FACS: Fellow, American College of Surgeons; MD: Doctor of Medicine; FETCS: Fellow of the European Board of Thoracic and Cardiovascular Surgeons

		n	%
Academic Degree	FACS	2	6.45
	FETCS	1	3.22
	MD	28	90.32
Country	France	15	48.38
	Italy	12	38.7
	Switzerland	1	3.22
	Taiwan	1	3.22
	United States	2	6.45
		Min-Max	Mean±sd (Median)
Time since upload (days)		1536-7181	4170.51±1819.5 (4248)
Run time (seconds)		81-1870	631.02±483.61 (492)
Number of views (n)		385-4885	1274.16±936.34 (965)
Number of likes (n)		2-123	23.61±26.86 (12)

A total of 31 videos about MIVAP returned by the WebSurg® search engine in reply to the keywords “MIVAP”, ‘’video assisted parathyroidectomy’’ and ‘’minimally invasive parathyroidectomy’’ were included in this study. 90.32% of the videos were uploaded by members with an academic degree of MD. 48.38% and 38.7% of the videos were uploaded by members from France and Italy, respectively.

The characteristics of the video scores are detailed in Table 6.

**Table 6:** Information on VPI, DISCERNqs, JAMABC, GQSc, and MIVAP-SS scores of the videos VPI: video power index; DISCERNqs: DISCERN questionnaire score; JAMABC: Journal of American Medical Association benchmark criteria; GQSc: global quality score; MIVAP-SS: Minimally invasive video-assisted parathyroidectomy scoring system

	Min-Max	Mean±sd (Median)
VPI	0.037-8.007	1.087±1.865 (0.264)
DISCERNqs	26-72	42.51±14 (38)
JAMABC	2-3	2.25±0.43 (2)
GQSc	1-5	3.09±1.08 (3)
MIVAP-SS	4-15	10.03±2.72 (10)

The mean VPI, DISCERNqs, JAMABC, GQSc, and MIVAP-SS scores of WebSurg® content on MIVAP were 1.087±1.865, 42.51±14, 2.25±0.43, 3.09±1.08, and 10.03±2.72, respectively.

The VPI, DISCERNqs, JAMABC, GQSc, MIVAP-SS scores of the videos uploaded by the members were evaluated according to academic degree and country, and a significant difference was found only between the country and VPI scores (p=0.045) (Table 7).

**Table 7:** Comparison of scores according to descriptive characteristics FACS: Fellow, American College of Surgeons; MD: Doctor of Medicine; FETCS: Fellow of the European Board of Thoracic and Cardiovascular Surgeons; VPI: video power index; DISCERNqs: DISCERN questionnaire score; JAMABC: Journal of American Medical Association benchmark criteria; GQSc: global quality score; MIVAP-SS: Minimally invasive video-assisted parathyroidectomy scoring system

	VPI	DISCERNqs	JAMABC	GQSc	MIVAP-SS
Academic degree					
FACS	0.21±0.24 (0.21)	52.50±27.58 (52.50)	2.00±0.00 (2)	3.50±2.12 (3.50)	10.00±1.41 (10)
FETCS	0.24	40	3	3	12
MD	1.18±1.98 (0.34)	41.89±13.74 (38)	2.25±0.44 (2)	3.07±1.09 (3)	9.96±2.89 (10)
p	0.511	0.788	0.177	0.977	0.662
Country					
France	1.91±2.48 (0.53)	37.87±9.64 (38)	2.20±0.41 (2)	2.87±0.83 (3)	9.73±2.58 (10)
Italy	0.23±0.17 (0.19)	42.08±15.08 (37.50)	2.33±0.49 (2)	3.00±1.28 (3)	10.25±3.17 (10.50)
Switzerland	0.09	57	2	4	10
Taiwan	0.38	72	2	5	11
United States	0.90±1.01 (0.90)	58.00±19.80 (58)	2.50±0.71 (2.50)	4.00±1.41 (4)	10.50±4.95 (10.50)
p	0.045	0.104	0.761	0.261	0.983

The correlations between time since upload, run time, number of views and likes versus video assessment scores were evaluated (Table 8).

**Table 8:** Determination of the relationship between quantitative variables and scores VPI: video power index; DISCERNqs: DISCERN questionnaire score; JAMABC: Journal of American Medical Association benchmark criteria; GQSc: global quality score; MIVAP-SS: Minimally invasive video-assisted parathyroidectomy scoring system

		VPI	DISCERNqs	JAMABC	GQSc	MIVAP-SS
Time since upload (days)	r	-0.683	-0.251	-0.157	-0.270	-0.170
	p	<0.001	0.174	0.400	0.141	0.361
Run time (seconds)	r	-0.054	0.518	0.528	0.510	0.596
	p	0.774	0.003	0.002	0.003	<0.001
View	r	0.078	-0.094	-0.165	-0.005	-0.077
	p	0.676	0.613	0.376	0.977	0.682
Like	r	0.927	0.139	0.342	0.193	0.316
	p	<0.001	0.454	0.059	0.297	0.083

The Spearman correlation analysis was performed between all indicators. While there was a negative correlation between time since upload and VPI score (r=-0.683, p<0.001), there was no significant relationship between time since upload and other video assessment scores (all p>0.05). There was a statistically significant positive correlation between the run time versus DISCERNqs, JAMABC, GQSc, and MIVAP-SS scores (p=0.003, p=0.002, p=0.003, p<0.001, respectively), whereas no significant correlation was noted among the run time and VPI score (p=0.774). No statistically significant differences were seen in video assessment scores according to the number of views (all p>0.05). The number of likes correlated strongly with the VPI score (r = 0.927; p<0.001), whereas there was no significant relationship between the number of likes and other video assessment scores (all p>0.05).

The correlation between the VPI, DISCERNqs, JAMABC, GQSc, and MIVAP-SS score values was examined (Table 9).

**Table 9:** Determination of the relationship between scores VPI: video power index; DISCERNqs: DISCERN questionnaire score; JAMABC: Journal of American Medical Association benchmark criteria; GQSc: global quality score; MIVAP-SS: minimally invasive video-assisted parathyroidectomy scoring system

		VPI	DISCERNqs	JAMABC	GQSc	MIVAP-SS
VPI	r	1.000				
	p	-				
DISCERNqs	r	0.256	1.000			
	p	0.164	-			
JAMABC	r	0.379	0.634	1.000		
	p	0.035	<0.001	-		
GQSc	r	0.312	0.927	0.617	1.000	
	p	0.088	<0,001	<0.001	-	
MIVAP-SS	r	0.389	0.727	0.739	0.778	1.000
	p	0.031	<0.001	<0.001	<0.001	-

**Figure 1:**
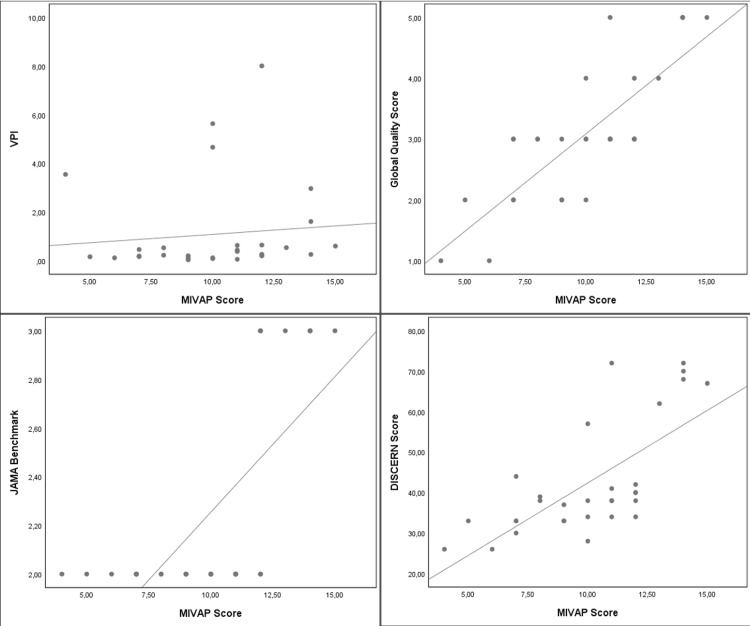
Scatter plots depicting the correlation between MIVAP-SS and other scoring systems MIVAP-SS: MIVAP scoring system

**Figure 2:**
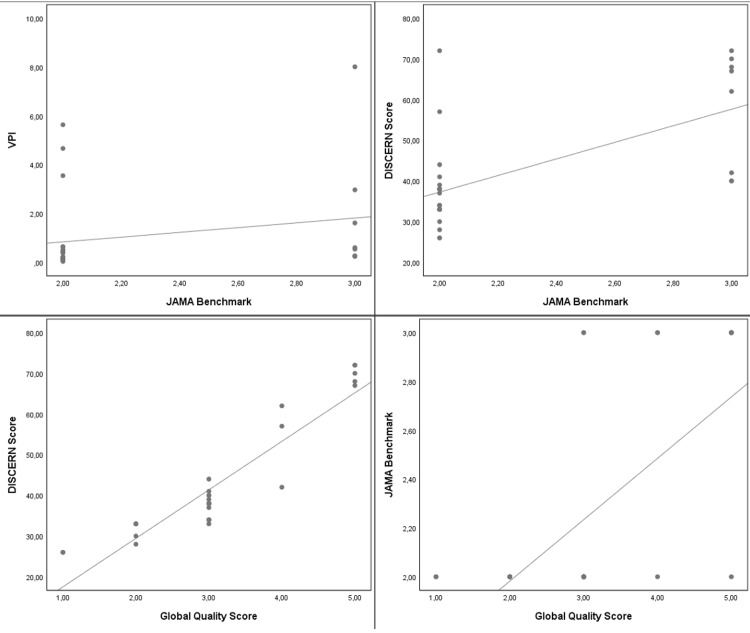
Scatter plots showing the correlation between the VPI, DISCERNqs, JAMABC, and GQSc scoring systems VPI: video power index; DISCERNqs: DISCERN questionnaire score; JAMABC: Journal of American Medical Association benchmark criteria; GQSc: global quality score

For the VPI score, the Spearman correlation analysis demonstrated a statistically significant positive correlation with the JAMABC (r=0.379, p=0.035) and MIVAP-SS scores (r=0.389, p=0.031). The JAMABC, GQSc, and MIVAP-SS scores had a strong, positive correlation for the DISCERNqs score (r=0.634, r=0.927, r=0.727, respectively, and all p<0.001). The GQSc and MIVAP-SS scores had a strong, positive correlation for the JAMABC score (r=0.617, r=0.739, respectively, and both p<0.001). The GQSc score also demonstrated a strong, positive correlation for the MIVAP-SS score (r=0.778, p <0.001). Scatter plots are used to observe the relationship between the VPI, DISCERNqs, JAMABC, GQSc, and MIVAP-SS scores (Figures 1-2).

## Discussion

Minimally invasive techniques have experienced a rise in popularity in recent years and have better results than conventional techniques, including better visualization of anatomical aspects and fewer postoperative complications. However, these advancements require a prolonged learning curve and make training more challenging for inexperienced surgeons. Thus, surgical education and technical skills training continue throughout the whole of surgeons’ lives. Different online learning video platforms, including WebSurg®, are concerned with both traditional surgical principles and current high-quality, new surgical techniques. They provide surgeons with valuable, clinical domain-specific, high-quality educational material with access to a wide variety of video content. Simulation-based medical education and instructional surgery videos have potentially positive effects on costs spent on training surgeons, and patients’ outcomes, including operative times and hospital stay [17-18]. WebSurg® provides surgeons with laparoscopic and endoscopic surgery videos, preoperative and postoperative videos that contribute to surgical training, and first-rate educational content, including expert feedback and lectures provided by experts from different countries in all fields of minimally invasive surgery.

Several different surgical approaches have been described in the surgical treatment of hyperparathyroidism. Bilateral neck exploration and minimally invasive parathyroidectomy are the most commonly used methods in parathyroid surgery. Minimally invasive parathyroidectomy includes minimally invasive open techniques and minimally invasive endoscopic techniques (video-assisted or fully endoscopic) [19]. Minimally invasive techniques have replaced bilateral neck exploration in patients with localized disease, as it is a safe procedure with lower morbidity, better cosmetic outcomes, higher cure rates, and cost-effectiveness. However, the standard approach is bilateral neck exploration in patients with failed localization studies, hereditary disease, concomitant thyroid disease, or suspected cancer [20]. Since the lateral endoscopic approach has 3 trocar entry sites, requires CO2 insufflation, and carries the risk of hypercapnia, respiratory acidosis, subcutaneous emphysema, and air embolism, is not preferred. On the other hand, remote access endoscopic parathyroidectomy requires a wide dissection area and has a long operation time and learning curve. However, remote access endoscopic parathyroidectomy techniques for parathyroid adenomas located in the mediastinum have significant advantages over conventional surgery. Among the currently used methods of MIP, MIVAP is the most frequently selected endoscopic technique. It is applied over the suprasternal notch, with a skin incision of approximately 1.5 cm in the midline, without requiring CO_2_ insufflation, and has a shorter learning curve and operative time compared to the total endoscopic approach [21-22]. In addition, providing the ability to perform a neck exploration and simultaneous en bloc thyroid lobectomy without the need for conversion to standard cervicotomy are the other advantages of this method [12].

Surgical videos are highly effective educational tools that enhance surgeons’ learning. Watching videos of the procedure is one of the usual steps of a surgeon’s learning. Parathyroidectomy training can be done safely with a short learning curve and good results. Video-assisted techniques also contribute to the learning process by understanding critical anatomic landmarks better.

WebSurg® offers the possibility to watch many different videos and lectures by members from different types of academic degrees. In this study, the greatest number of videos were uploaded by medical doctors (MDs). Contributors from all over the world send videos to WebSurg® in order to improve public health and share their experiences. This study also showed that members from France were a large contributor to the videos related to MIVAP, followed by Italy and United States.

In the present study, the mean DISCERNqs, JAMABC, GQSc, and MIVAP-SS scores of WebSurg® content on MIVAP were 42.51±14/80, 2.25±0.43/4, 3.09±1.08/5, and 10.03±2.72/25, respectively. These scores rates indicate that the reliability, transparency, and quality of WebSurg® videos about MIVAP are sufficient but below the expected quality corroborates previous studies [23-25]. No significant difference was observed between DISCERNqs, JAMABC, GQSc, and MIVAP-SS scores of the videos uploaded by the members in terms of academic degree and country. This means that WebSurg® videos associated with MIVAP are similar in educational quality, transparency, and accuracy. The reason for the insignificance may be related to the fact that the videos are uploaded by academic professionals, evaluated by a review committee, and go through a filtration process before being uploaded [26]. However, videos uploaded by members from France and the United States had higher popularity (VPI score) than videos uploaded by other countries. This may be attributed to the fact that most of the WebSurg® visitors originating from the United States and France [27].

The success of minimally invasive parathyroid surgery depends on multiple factors, including knowledge of parathyroid embryology and anatomy, variation in the size and location of the parathyroid glands, and surgical strategy. Multiple studies have shown that parathyroid surgery has very high cure rates (95-100%) and low morbidity rates (0.5-3%) as long as it is performed by experienced surgeons [28]. Surgeons performing a high volume of operations have shorter learning curves, but still, no consensus has been reached on learning curves for minimally invasive endoscopic parathyroidectomy techniques. The absence of tactile sensation, restricted surgical field, and unfamiliarity of the surgeon may also increase this learning curve. Berti et al. evaluated 185 patients who underwent MIVAP and reported that the operative time and postoperative complication rate of MIVAP decreased significantly with increasing experience [29].

In this study, there was a positive association between the DISCERNqs, JAMABC, GQSc, MIVAP-SS scores, and the run time of the videos. The run time of high educational and technical quality videos was longer because adequate time is required to present the data requested by the criteria in scoring systems. Considering the preoperative, perioperative, and postoperative technical assessments of the MIVAP procedure, there was a positive correlation between the MIVAP-SS score and VPI, DISCERNqs, JAMABC, and GQSc scores. This suggests that the videos are useful for surgical trainees to learn the procedure step-by-step before the surgery they will be performing. This article should also be of interest to surgeons-in-training in the area of endocrine surgery or head and neck surgery in times of coronavirus disease (COVID-19). The COVID-19 pandemic has led to the suspension of face-to-face educational activities to prevent the spread of the virus and mitigate its impact. Thus, it has given rise to the deployment of distance learning modalities, including video-based learning. The main limitation of this study was that only videos returned by the WebSurg® search engine were evaluated; other health-related websites were excluded from the study. Despite this limitation, the contributions and implications of the study are noteworthy; our study includes a comprehensive analysis that includes the interaction of all variables with many factors that may be relevant to the quality and accuracy of the videos.

## Conclusions

Despite the widespread use of the Internet, there are still debates about the quality of online platforms. WebSurg® can be used as a high-quality educational platform to inform surgeons about new technologies and techniques. The fact that all videos in WebSurg® are uploaded by health professionals after scientific filtration also increases the educational quality but the videos about the MIVAP procedure still remain below the expected quality. Our study showed that there is a positive relationship between the MIVAP-SS score and known scoring system scores. It is recommended to use MIVAP-SS points for standardizing MIVAP videos. Besides, the descriptive results show that the most popular videos are those newly uploaded, and to achieve high quality, the run time must be long enough to convey verbally or in writing what the criteria of the scoring systems require.
